# Neuronal photoactivation through second-harmonic near-infrared absorption by gold nanoparticles

**DOI:** 10.1038/s41377-018-0103-0

**Published:** 2018-12-05

**Authors:** Wieteke D. A. M. de Boer, Jan J. Hirtz, Antonio Capretti, Tom Gregorkiewicz, Mercè Izquierdo-Serra, Shuting Han, Christophe Dupre, Yuriy Shymkiv, Rafael Yuste

**Affiliations:** 1 NeuroTechnology Center, Department of Biological Sciences, Columbia University, New York, NY 10027 USA; 2 Van der Waals–Zeeman Institute, University of Amsterdam, 1098 XH Amsterdam, Netherlands; 3 Present Address: Laboratori de Fisiologia Molecular, Departament de Ciències Experimentals i de la Salut, Universitat Pompeu Fabra, Barcelona, Spain

## Abstract

Optical activation of neurons requires genetic manipulation or the use of chemical photoactivators with undesirable side effects. As a solution to these disadvantages, here, we demonstrate optically evoked neuronal activity in mouse cortical neurons in acute slices and in vivo by nonlinear excitation of gold nanoparticles. In addition, we use this approach to stimulate individual epitheliomuscular cells and evoke body contractions in *Hydra vulgaris*. To achieve this, we use a low-power pulsed near-infrared excitation at the double-wavelength of the plasmon resonance of gold nanoparticles, which enables optical sectioning and allows for high spatial precision and large penetration depth. The effect is explained by second-harmonic Mie scattering, demonstrating light absorption by a second-order nonlinear process, which enables photothermal stimulation of the cells. Our approach also minimizes photodamage, demonstrating a major advancement towards precise and harmless photoactivation for neuroscience and human therapeutics.

## Zapping neurons to life with infrared light

Neuronal impulses can be generated by aiming a near-infrared laser beam at gold nanoparticles precisely tethered to brain cells. Wieteke de Boer and Jan Hirtz of the NeuroTechnology Center at Columbia University, USA, and colleagues developed the technique, which could provide a nontoxic, nongenetic alternative to commonly used optical methods for activating brain cells. They tested it in live mouse brain tissue, and also used it to induce movement in a tiny freshwater animal called *Hydra vulgaris*. The authors demonstrate the potential for targeted stimulation of neurons through the nonlinear absorption of light by nanoparticles. By using low-power short-pulsed near-infrared excitation, the photodamage of the tissue is minimal. The approach shows promise for application in biological systems and for future treatments of neurological and mental disorders.

## Introduction

The interaction of light with metal nanoparticles (NPs) has been of great interest for both fundamental research and a wide variety of applications^1–3^. The optical spectra of noble metals are dominated by the resonant coupling of the incident field with the collective oscillations of conducting electrons, known as surface plasmon resonance. For metallic NPs, the excitation of the surface plasmons is confined to their curved geometries, resulting in the so-called localized surface plasmon resonance (LSPR). The spectral position of the LSPR highly depends on the dielectric properties, the size and the shape of the particles^4^, as well as on the dielectric constant of the host matrix, due to the screening of the Coulomb attraction between the oscillating electrons. In this way, nanocomposites can be accurately designed such that the LSPR precisely matches a desired application^5–7^.

After optical excitation, the LSPR relaxes through electron−electron and electron−phonon scattering within a typical time scale of 0.1–1 ps, after which the heat generated inside the NP is transferred to the surroundings of the metallic structure on a longer time scale ranging from 100 ps to 10 ns via thermal conduction. The large optical absorption cross-section of these NPs (generally 100–1000× larger than that of standard fluorophores^8^) makes them ideal candidates as efficient nanosources of heat. In particular, gold nanoparticles (Au NPs) have attracted much attention for biological applications, e.g., photothermal cancer therapy^9^ and drug delivery^10^, due to their attractive characteristics, such as high photostability, low toxicity, and the absence of observed pharmacological side effects. Recently, Au NPs were used in neuroscience to evoke action potentials (APs) in neurons of mouse dorsal root ganglion cell cultures and brain slices^11^. This was achieved through excitation of the NPs, increasing the local temperature. In this case, the authors used light in the visible regime centered at the LSPR feature under semicontinuous wave conditions. However, excitation wavelengths in the visible range have limited applicability for living tissue, as the penetration depth of light in vivo at these wavelengths is rather small (<100 µm) for excitation powers below the tissue-damaging threshold. This hurdle can be overcome by using near-infrared (NIR) excitation light, which penetrates up to several mm due to the low absorption/scattering cross-sections for tissue at these wavelengths, rendering it in addition far less photo damaging, even at high excitation powers. Moreover, femtosecond (fs)-pulsed NIR lasers can generate optical sectioning due to nonlinear absorption processes. This is employed, e.g., in two-photon microscopy and two-photon optogenetics, rendering these methods extremely suitable for in vivo applications^12^ and allowing single-cell resolution imaging and photoactivation^13^. In fact, previous studies have shown that Au NPs and Au nanorods can be efficiently excited using fs-pulsed NIR excitation^14–16^. In the case of Au nanorods, NIR wavelengths are absorbed through linear absorption, as these particles feature a (longitudinal) plasmon mode, which generally lies in the NIR spectral regime. Although linear absorption is generally more efficient than a nonlinear absorption process, it has limited spatial resolution (i.e., due to the excitation of the specimen throughout the entire excitation pathway) and is therefore not ideal for high-precision targeted experiments in biological systems. In the case of the small Au NPs used in this study, the absorption of NIR excitation light in the specimen is dominated by a second-order nonlinear process occurring in the NPs, where a fraction of the excitation light is converted into a second-harmonic (SH) component^17,18^; hence, high spatial resolution is achieved. Following this approach, we used small spherical Au NPs for nonlinear photoactivation of mouse neurons in slices and in vivo and epitheliomuscular cells of *Hydra vulgaris* with high spatial resolution. We quantify the excitation energy absorbed by the NPs using the extension of Mie scattering to the SH regime. Subsequently, we model the spatial and temporal temperature dependence by using a heat diffusion model for pulsed excitation sources. This technique, using fs-pulsed NIR excitation of Au NPs, is in many aspects advantageous compared to currently available methods to optically activate neurons.

## Results

### Photoactivation of neurons in cortical brain slices

**Fig. 1:**
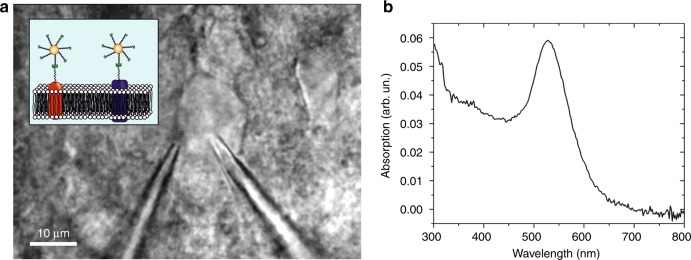
Experimental strategy and Au NP absorption spectrum. **a** Bright-field microscope image of a patch-clamped layer 5 neuron, with the patch-clamp pipette (right) and the NP application pipette (left). Inset: schematic illustration of NPs tethered to the membrane through streptavidin-biotin binding. The biotin adheres to the membrane through conA (which binds to specific terminal sugar residues found in sugars, glycoproteins and glycolipids) or NHS (which binds to lysine-based membrane proteins). **b** Absorption spectrum of the NPs (~0.1 nM) in a spectral window of 300−800 nm, with an absorption feature centered at approximately 525 nm. The absorption peak is related to the surface plasmon resonance, which is a resonant oscillation of free electrons at the interface between a negative and positive permittivity material upon interaction with light

**Fig. 2:**
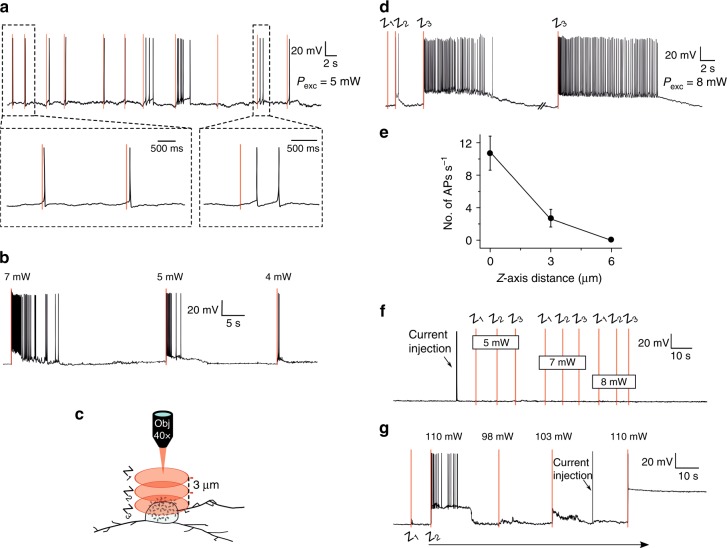
Nonlinear photoactivation of neurons with Au NPs in brain slices. **a** Current clamp recording of a whole-cell patch-clamped layer 5 neuron from acute mouse cortical slices after application of conA and Au NPs. The recording shows a consistent response to 5 mW laser excitation of either a single or a few APs per stimulation. The red-shaded area depicts the time of the laser stimulation, and also applies to all following panels. **b** Recording in a slice incubated with the NHS-biotin linker. The first stimulation with 7 mW excitation power evokes an intense burst of APs. Lowering the power to 5 and 4 mW reduces the firing rate. **c** Schematic illustration of the excitation at the different focal planes. **d** Recordings performed with optical stimulations of 8 mW at different positions along the *Z*-axis with respect to the cell soma position (*Z*_1_ = 6 µm, *Z*_2_ = 3 µm, and *Z*_3_ = 0 µm). **e** *Z*-axis distance-dependent AP firing rate (*n* = 12, NHS-incubated cells). The error bars depict the standard error of the mean. **f** Control experiment without NPs. The responsiveness of the neuron was first confirmed by current injection (400 pA for 5 ms), after which the stimulation protocol was performed at three different *Z*-positions with respect to the cell soma (*Z*_1_, *Z*_2_, and *Z*_3_ same as in **c**, **d**). No APs were evoked. **g** Using high excitation power (at 1040 nm) to evoke responses with direct NIR excitation (no Au NPs present). After the first intense AP burst (at *Z*_2_ with 110 mW), the power is adjusted. Repeating the excitation with 110 mW results in cell death. The responsiveness of the neuron is checked by injecting a current pulse (400 pA for 5 ms)

We first performed photostimulation experiments on layer 5 pyramidal neurons from acute brain slices of the mouse cortex. The membrane potential was monitored by simultaneous whole-cell patch-clamp recordings. Streptavidin-functionalized Au NPs were immobilized by tethering them onto neuronal membranes coated with a concanavalin A (conA)-biotin complex (see the inset of Fig. 1a for a schematic illustration—see the Materials and Methods section for details). Alternatively, slices were incubated with an NHS-biotin linker prior to the recording. Figure 1a shows a bright-field image of the NP application process. For optical stimulation, the laser was set to 1040 nm, close to the double-wavelength of the plasmon resonance of the Au NPs (see Fig. 1b for an absorption spectrum of the sample). Figure 2a shows the response to a spiral-shaped scanning pattern with 5 mW excitation on sample, where almost all photostimulations evoke a single AP or a few APs. By changing the excitation power of the stimulation, we could accurately tune the intensity of the response, where a difference of as little as 3 mW could strongly alter a response—see Fig. 2b, where a stimulation of 7 mW evokes an intense response, whereas lowering the excitation power to 4 mW results in a moderate response of a few APs. Changing the focal plane of the laser with respect to the cell soma demonstrated optical sectioning consistent with a nonlinear excitation (Fig. 2c): Moving the laser focus 6 µm away from the cell soma in the Z-plane eliminated the AP response. Figure 2d shows a recording where moving in steps of 3 µm towards the soma resulted first in a change from nonresponsive to a few APs and after the second step to an intense burst of APs (see Fig. 2e for statistics). Recordings could be as long as 1 h after the first stimulation without affecting the health of the cell. We were able to successfully evoke APs repeatedly in 22 of 26 NHS-incubated cells, and all of 8 cells with conA application. Control experiments performed under identical excitation conditions without NPs did not result in any AP generation (Fig. 2f). However, APs could be evoked as a result of direct pulsed IR absorption^19^ using excitation powers 20–50× higher than in case of the NP stimulation experiment (Fig. 2g). As evidenced by the trace shown in Fig. 2g, excitation with such high powers (~100 mW) was hard to control and could easily result in permanent cell damage.

### Neuronal photostimulation in vivo

**Fig. 3:**
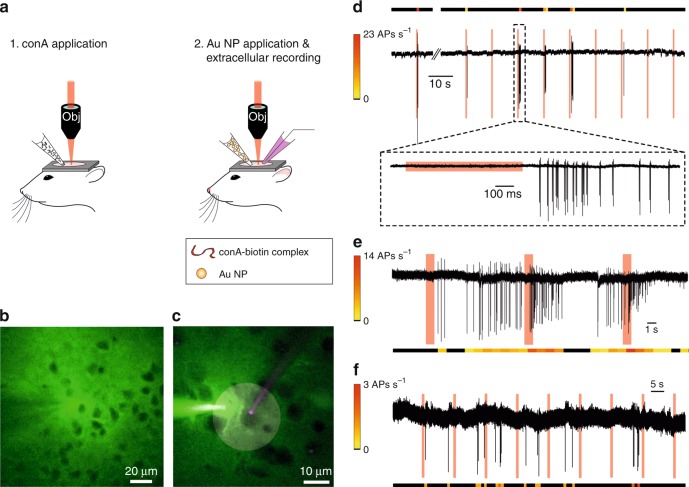
In vivo nonlinear photoactivation of cortical neurons with Au NPs. **a** Schematic illustration of the conA (left) and Au NP (right) application procedure. **b** Image of an injection of con A-biotin complex (with Alexa 488, green); cell somata can be identified as shadow-like features. **c** Image of a loose-seal patch-clamp recording using a pipette filled with Alexa 594 (magenta) containing ACSF and a pipette with streptavidin-coated NP solution (with Alexa 488, green), applied in close vicinity of the neuron prior to the stimulation experiment. The shaded circle represents the spiral stimulation pattern. **d** Extracellular recording of a layer 2/3 neuron showing a repeated response of multiple APs (see color bar for AP firing rate) to optical stimulations. The lower panel shows a zoom-in for more details. The red-shaded area depicts the time of the laser stimulation, and also applies to the following panels. **e** Different example of a recording of a mouse layer 2/3 neuron. Here, the intrinsic firing rate of the cell drastically increases as a result of the optical excitation. **f** Control experiment in which the spiral stimulation of a neuron was performed in the absence of NPs, demonstrating that the pulsed NIR excitation alone does not evoke APs. Only one AP was coincidentally observed during laser stimulation due to intrinsic AP firing

Next, we tested neuronal stimulation with NP excitation in vivo first in the visual cortex of anesthetized mice. Prior to optical stimulation, the conA-biotin complex mixed with Alexa Fluor 488 (green, for visualization) was pressure-injected into layer 2 of the visual cortex (see Fig. 3a for a schematic on the application procedure and Fig. 3b for a two-photon fluorescence image). Subsequently, a pipette with streptavidin- or neutravidin-coated NPs (and Alexa Fluor 488) was inserted in the vicinity of the injection site, and NPs were applied (Fig. 3a, c). Neuronal somata were visualized as shadows through the fluorescent signal of Alexa Fluor 488, enabling the subsequent establishing of targeted loose-seal patch-clamp recordings (Fig. 3c). Using 40 mW excitation power on the sample, we were able to evoke responses, either in the form of short bursts of APs directly following the stimulation (Fig. 3d) or increases in the intrinsic firing rate (Fig. 3e). In all but one case, we were able to induce AP responses more than once (*n* = 6). In control experiments in which no NPs were applied (Fig. 3f), we neither evoked APs nor observed any effect on the intrinsic firing rate of the stimulated cells (*n* = 7) under the same excitation conditions as those used in the NP-excitation experiments.

### Evoking body contractions in *Hydra*

**Fig. 4:**
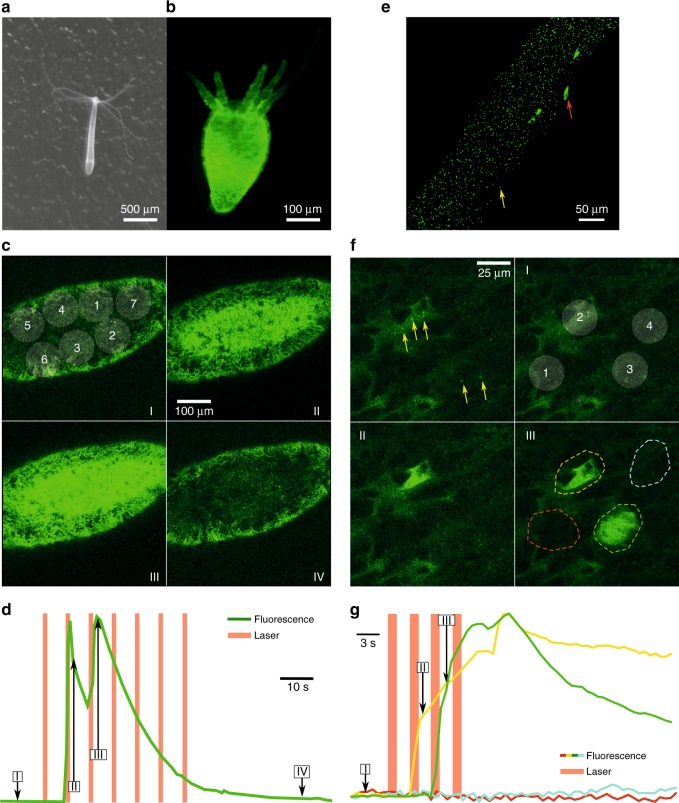
Simultaneous Ca^2+^ imaging and Au NP photoactivation in *Hydra* **a** Bright-field image of a *Hydra* specimen. **b** Fluorescent image of an Au NP-incubated *Hydra* expressing GCaMP6s in epitheliomuscular cells. **c** Example of systemic nonlinear excitation of a *Hydra*. Images of different frames in the synchronous fluorescence recording are shown (refer to **d** for the time scale). The white-shaded area depicts the stimulation patterns (20 mW on sample). The second and third stimulations evoked muscle activity. **d** The fluorescence signal (integrated over the entire image window) shows an intensity increase coinciding with the second and third stimulations. **e** Confocal microscope image of a *Hydra* incubated with Au NPs with a fluorescent tag (yellow arrow); larger green spots are epithelial cells (red arrow). **f** Single frames taken from a time-dependent fluorescence recording of epithelial cells of a transgenic GCaMP6s *Hydra* incubated with Au NPs with a fluorescent tag (DY488, green). The left upper panel shows the presence of NPs (yellow arrows) close to or on the cell membranes; cells can be distinguished as dark shadows haloed by green emission. The right upper panel (I) illustrates the excitation pattern used, with each circle representing a spiral excitation. The lower left panel (II) shows an image taken at the end of the second excitation. The last panel (III) corresponds to a frame directly after the third excitation. The colored dashed outlines indicate the area integrated to obtain the time-dependent fluorescence intensity, as shown in panel (**g**). Excitation power on the sample was 20 mW. **g** Fluorescence intensities of the areas corresponding to the second and third excitations (yellow and green, resp.) show an increase starting at the onset of the stimulation

For our next in vivo experiments, we aimed to demonstrate the capability of our method to control behavior. We used the cnidarian *H. vulgaris* (see Fig. 4a, b for a bright-field and fluorescence microscope image), which can serve as a model organism for circuit neuroscience^20,21^ expressing the calcium indicator GCaMP in epitheliomuscular cells. Optically evoking activity in neurons and muscle cells in invertebrates has been a powerful method to study the basis of neuronal circuits underlying behavioral patterns^22^. In the first series of experiments, animals were incubated overnight with streptavidin-coated Au NPs combined with gastric injection of Au NPs. *Hydra* were sedated prior to the stimulation experiment. We used a systematic stimulation protocol consisting of several consecutive spiral scanning patterns covering the entire body of *Hydra*, which resulted in strong changes in GCaMP fluorescence intensity, indicating muscular activity (see Fig. 4c, d). We also found that, in some cases, despite the sedation and immobilization by embedding them in 1% agarose, body contractions were evoked as a result of pulsed NIR laser stimulations (see Supplementary Materials online for the full time-dependent recording (1)). In a second set of experiments, we used NPs with a fluorescent tag (DY 488, green) to visualize the position of the NPs within the body of the animal (Fig. 4e). In experiments where we optically targeted individual epitheliomuscular cells either with or without NPs on their membrane (stimulation 2 and 3, and 1 and 4 in Fig. 4f, respectively), we observed increases in GCaMP fluorescence only when stimulating the cells on which we could localize NPs (Fig. 4f, g, and Supplementary Materials online for a full time-dependent recording (2)). Although not structurally investigated in this experiment, it should be noted that, due to the spectral overlap of the imaging laser with the second-harmonic feature of the NPs, the imaging laser could potentially have contributed to the NP excitation, by lowering the effective NP excitation laser power threshold to achieve optical activation of the cells. Repetitions of the excitation indicated that this stimulation method did not affect the viability and responsivity of the majority of the targeted cells (*n* = 6 from *n* = 8). In combination with the axial resolution experiments performed in brain slices, this demonstrates the high spatial precision achieved by nonlinear Au NP excitation.

In summary, we experimentally demonstrated optically evoked activity of mouse cortical neurons and epitheliomuscular cells in *Hydra* by low-power pulsed NIR excitation of Au NPs. We used simple, effective methods to tether the Au NPs to biological membranes, which allowed efficient, high-resolution stimulation of the targeted neurons. As mentioned, it has previously been shown that neuronal activity can be evoked indirectly through excitation of NPs using visible light, where the generation of plasmons induces changes in the local temperature^11^. Importantly, in the present study, the excitation is performed with fs-pulsed NIR light, where light absorption by the NPs occurs largely through a nonlinear second-order process, which enables tissue penetration and optical sectioning.

### Calculation of total absorbed energy

To understand the mechanisms underlying the nonlinear photoexcitation by the NPs, we explored our results numerically with a model. We first calculated the total absorbed excitation energy (i.e., linear and nonlinear contributions) per pulse. We used the extension of Mie scattering to the SH regime to obtain the linear and SH absorption cross-sections of our Au NPs^23,24^. Subsequently, the temperature profile in space and time was estimated using a thermodynamic model developed for pulsed optical excitation^25^.

**Fig. 5:**
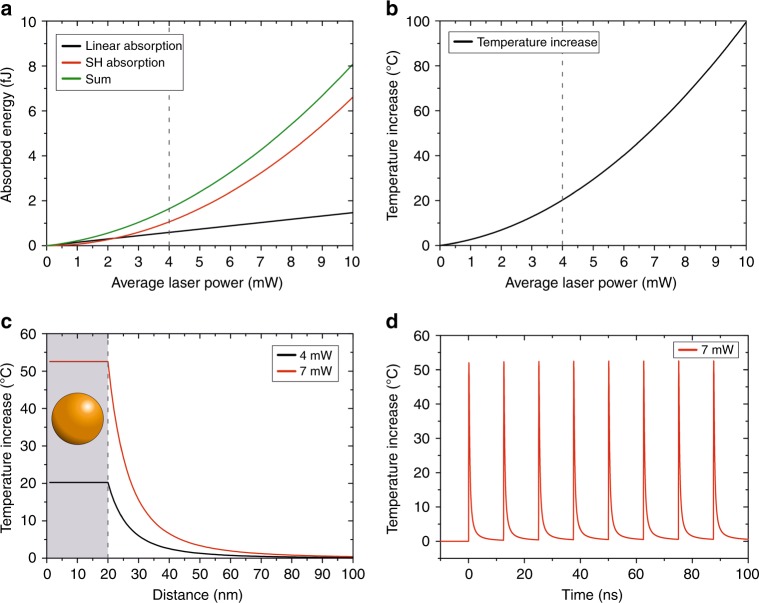
Excitation power and temperature dependence. **a** The absorbed energy per pulse as a function of the average power of the incident laser. The contribution of the linear absorption (black curve) and the SH nonlinear absorption (red curve) combined add up to the total absorption (green curve). **b** The temperature increase in the Au NP as a function of the average excitation power of the incident laser. The dotted line represents the minimum activation threshold as found in the experiments. **c** The temperature increase as a function of the distance from the NP surface (dotted line) for an average incident laser power of 4 mW (black line) and 7 mW (red line). The model assumes a homogeneous temperature distribution within the NP (shaded area). **d** Time-dependent temperature increase in a time window of 100 ns for an excitation power of 7 mW at the surface of the NP. Consecutive pulses (every 12.5 ns—laser repetition rate *f* = 80 MHz) at the same spot amount to an increase of ~0.5 °C

The (linear) absorption cross-section $C_{{\rm {abs}}}^{({\rm {NIR}})}$ is easily calculated through standard Mie theory (see Materials and methods). Conversely, the SH nonlinear contribution is not straightforward because there are several distinct sources of nonlinearities whose relative magnitudes are still undetermined^17,26–31^. While most of the relevant literature on SH generation from metal NPs focuses on the radiated field (i.e., on the SH scattering cross-section), here we are interested in the SH power *absorbed* within the NP, which is subsequently dissipated as heat. We define the absorption cross-section $C_{{\rm {abs}}}^{({\rm {SH}})}$ at the SH wavelength of an Au NP excited by a NIR plane wave with electric field amplitude $E_0^{({\rm {NIR}})}$ by:

1 $$C_{{\rm {abs}}}^{({\rm {SH}})} = k_0^{({\rm {SH}})}{\int\int\int}_V {\varepsilon _2^{({\rm {SH}})}\frac{{|E^{({\rm {SH}})}|^2}}{{|E_0^{({\rm {NIR}})}|^2}}{\rm d}V}$$

where $\varepsilon _2^{({\rm {SH}})}$ is the imaginary permittivity of gold at the SH wavelength, $k_0^{({\rm {SH}})}$ is the SH wavenumber and $E^{({\rm {SH}})}$ is the electric field amplitude across the NP, calculated using the analytical model developed in ref. ^23^. We base our calculations on the sources of nonlinearities experimentally estimated in ref. ^27^, as this was performed at the same excitation wavelength *λ*_NIR_=1040 nm. Figure 5a shows the contribution of the linear and second-order absorption components and the total absorbed energy for the excitation power range used in the cortical brain slice experiments, where the linear, SH, and the total absorption are shown by black, red, and green lines, respectively. Due to the quadratic dependence of the second-order absorption on excitation power, the contribution of this part to the total absorbed energy for the excitation regime used in this study (>4 mW, see dotted line in panel (a)) is the most significant^32^.

### Calculation of the local temperature increase

After quantifying the total absorbed energy, we calculated the temperature increase inside the Au NPs, as shown in Fig. 5b. The generated heat subsequently dissipates to the surrounding medium, as shown in Fig. 5c for two different excitation powers. The temperature decays according to *T* α *r*^−^^3^ (outside the NP); hence, the temperature increase decays to practically zero within ~100 nm for both excitation powers. As we use short linkers for tethering the Au NPs onto the cell membrane (~15 nm, partially dependent on their elasticity), the average temperature increase for 4 mW is approximately 5 °C. This is in agreement with previously reported threshold values required for evoking neuronal activity^11^. For higher excitation laser powers, the temperature increases at ~15 nm from the surface of the NP are higher (on the order of ~10 °C at an ~15 nm distance from the NP surface). From Fig. 2, it is evident that these excitation powers induce a more intense response.

Exposure to temperatures higher than physiological values can easily have noxious effects in biological tissue. In our case, the use of small spherical Au NPs in combination with pulsed fs excitation limits these damaging effects for multiple reasons: Firstly, as shown in Eq. (5) of Materials and methods, the spatial temperature dependence follows the relation *T* ∝ *r*^−3^, resulting in strong localization of the temperature increase—as opposed to the temperature dependence for continuous-wave excitation, which decays according to *T* ∝ *r*^−1^. Therefore, specific segments or components of the cell can be targeted with high accuracy. Second, in our experiments, we use a spatially moving excitation spot, which evidently reduces the probability of cumulative thermal damage. To confirm that this damage is indeed absent, we simulated the temperature increase at the surface of the NP for a multitude of consecutive excitation pulses at the same location at one of the higher power excitations used in the experiments performed on brain slices (i.e., 7 mW) in a 100 ns time window. The (approximative) temporal temperature dependence with pulsed excitation is expressed by^25^:

2 $$T(t) = \frac{{E_{{\rm {abs}}}}}{{c_{\rm w}\rho _{\rm w}\left( {4\pi a_{\rm w}t} \right)^{3/2}}}e^{ - (R_{{\rm {NP}}}^2/(4\pi a_{\rm w}t))}$$

where $a_{\rm w}$is the thermal diffusion constant of water ($0.143 \times {10}^{ - 6}\, {{\rm m}}^{2}\, {{\rm s}}^{-1}$). The trend shows that, when multiple pulses with this excitation power are absorbed by the same NP, it leads to a temperature increase of ~0.5 °C at the surface of the NP within this time window (Fig. 5d). Consequently, the residual temperature increase at the cell surface will be practically zero. In addition, a spatially movable pulsed excitation source also allows for the generated heat to quickly dissipate in the surrounding (unexcited) tissue.

The increase in temperature arising from the NIR excitation of Au NPs could in principle be measured directly by temperature-sensitive compounds. For example, nanodiamonds with nitrogen vacancy centers have been demonstrated to be ultrasensitive temperature sensors in human embryonic fibroblasts^33^. However, it is reasonable to expect this technique would be challenging in our experimental configuration, as this approach generally uses (semi)continuous-wave photoexcitation to induce (semi)steady-state temperature changes, as opposed to our experimental conditions. This promising route can be the subject of future investigations.

## Discussion

Here, we demonstrate, using a combined experimental and theoretical approach, that NIR Au NP stimulation presents an attractive method to optically control neuronal activity, lacking some of the drawbacks and limitations of currently available photostimulation techniques. One commonly used optical technique for photostimulation is optogenetics, which utilizes expression of photosensitive membrane channels in cells in order to make them photoactivatable^34,35^. In recent years, advances in two-photon optogenetics have been made^36–41^, even enabling the imprinting and recalling of activity patterns into the cortex of awake behaving mice^13^. However, due to the need for genetic modification, employing optogenetics usually requires a substantial lead time and can only be applied to animal models where genetic modifications are standard. As an alternative, photolyzing caged glutamate via two-photon excitation does not require genetic modification and can also achieve AP generation, yet the required concentrations needed for two-photon stimulation can have the unwanted side effect of blocking a substantial fraction of GABAergic transmission^42^. Recently developed methods using azobenzene-based photoswitches offer yet another approach to optically control neuronal activity, but the need for high light intensities limits their use for deep tissue applications^43^. These considerations render these techniques impractical for application in human patients as practical therapeutic tools to control neuronal activity^44,45^. Au NPs, on the other hand, possess none of these limitations, as they do not require genetic modification, are nontoxic even in high concentrations^46^ and are extremely effective even in small quantities. In addition, their optical properties (e.g., absorption wavelength and cross-section) can be precisely adjusted to match experimental requirements by simply changing NP size, shape, and configuration^47–49^. Furthermore, their surface can be (bio)chemically modified, enabling implementation in or tethering to virtually any type of biological tissue and allowing them to pass through the blood−brain barrier for easy delivery^50^. These features, in combination with their exceptional intrinsic properties, such as high photostability and high excitation efficiencies^51,52^, render them extremely suitable for application in biological systems and even potentially for human therapy and future treatments of neurological and mental disorders.

In conclusion, we show that we can successfully photoactivate neurons in the mouse cortex both in vitro and in vivo using pulsed NIR low-power excitation of Au NPs. NP-targeted stimulation can even trigger behavioral responses, as demonstrated by the body contractions evoked in *Hydra*. Based on our analysis, we conclude that the effect is a result of excitation energy absorption mediated by the second-order nonlinear optical response of plasmonic NPs, which provides great spatial accuracy and low photodamage. Although the photoactivation of neurons with Au NPs has been previously demonstrated with visible light, to our knowledge, we are the first to perform these stimulations in a nonlinear regime with NIR light sources and employ this technique successfully in an in vivo configuration. Our study demonstrates that the use of Au NPs in biological systems provides an attractive, nontoxic, nongenetic alternative to commonly used optical methods for evoking neuronal activity.

## Materials and methods

### Acute slice preparation and surgical procedures for mouse in vivo experiments

Animal handling and experimentation were performed according to the US National Institutes of Health and Columbia Institutional Animal Care and Use Committee guidelines. For acute slice experiments, coronal sections of the neocortex of postnatal days 7–12-old C57BL/6 mice of both sexes were prepared using a Leica VT1200S vibratome. The animal was decapitated, and the brain was quickly removed. Slices of 300 µm thickness were prepared in ice-cold slicing solution^53^ containing (in mM): 93 *N*-Methyl-d-glucamine, 2.5 KCl, 1.2 NaH_2_PO_4_, 30 NaHCO_3_, 20 4-(2-hydroxyethyl)piperazine-1-ethanesulfonic acid (HEPES), 25 glucose, 5 Na-ascorbate, 3 Na-pyruvate, 10 MgSO_4_, and 0.5 CaCl_2_. The pH was adjusted with HCl to 7.3 and the solution was bubbled with 95% O_2_ and 5% CO_2_. After a short recovery period (~4 min) in 35–37 °C warm slicing solution, slices were kept at room temperature in artificial cerebrospinal fluid (ACSF) containing (in mM): 126 NaCl, 26 NaHCO_3_, 1.145 NaH_2_PO4, 10 glucose, 3 KCl, 0.1 Na-pyruvate, 0.4 Na-ascorbate, 2 MgSO_4_, and 2 CaCl_2_. The osmolarity was ~300 mOsm and the solution was bubbled with 95% O_2_ and 5% CO_2_ until being transferred into a recording chamber. In vivo experiments were carried out on C57BL/6 postnatal day 50–70 mice of both sexes. Mice were anesthetized with isoflurane (1.5–2% partial pressure in air). A small flap of skin above the skull was removed, and a titanium head plate with a central foramen (7 × 7 mm) was attached to the skull with dental cement above the left hemisphere. Then, a craniotomy was performed in the region of the visual cortex. A quadratic section of the skull was thinned using a dental drill until a small piece (approximately 2 mm in diameter) of skull could be removed effortlessly with fine forceps. The dura mater was kept intact.

### Generation of transgenic *Hydra*

*Hydra vulgaris* were maintained in the dark at 18 °C and were fed freshly hatched *artemia nauplii* once a week or more frequently when the colony needed to grow. Transgenic lines were created according to ref. ^54^. Accordingly, Hydra oocytes were injected with DNA (2.5 mg/ml) through a micropipette made with a pipette puller (P-97, Sutter Instruments Co., CA, USA) from borosilicate glass pipettes and held with a microinjector (IM-9B, Narishige, NY, USA) controlled with a joystick micromanipulator (MN-151, Narishige, NY, USA).

The injected DNA was a modified version of the pHyVec1 plasmid (#34789, Addgene, MA, USA) where we replaced the GFP sequence (found between the *pst*l and *Eco*RI restriction sites) with a GCaMP6s sequence that was codon-optimized for *Hydra* (DNA2.0, Menlo Park, CA, USA). Just before the injection, 10 µl of DNA (2.5 mg/ml) were added to 6 µl of phenol red and centrifuged for 10 min at 14,800 rpm to collect the debris at the bottom of the tube. Once the eggs were injected, they were placed in the dark at 18 °C for 2 weeks and then returned to room temperature. After a few days, eggs started hatching, and the young hatchlings were fed for a couple of days before being screened for transgenic epithelial cells. Transgenic *Hydra* generated in this way are mosaic. Therefore, we kept these animals growing and asexually reproducing and selected the offspring according to the number of epithelial cells that were expressing the transgene (a procedure known as clonal propagation). We repeated this procedure until the amount of expression reached a maximum. For the selection of the transgenic GCaMP6s *Hydra* with the highest level of expression in their epithelial cells, the fluorescence of the specimen was imaged (BX50W1, Olympus, PA, USA) using a 488 nm diode laser as the excitation source (OBIS 488-60-LS, Coherent, CA, USA) in combination with a confocal scanner (Ultraview, PerkinElmer, OH, USA), guided through a ×20/0.5NA objective (Olympus, PA, USA) and registered with a scientific CMOS camera (Orca-Flash4.0, Hamamatsu, Japan).

Based on this procedure, each transgenic cell is expected to have the same genome since only one injection of the plasmid occurred and every animal was obtained from the same original individual by asexual reproduction. Additionally, the expression is driven by an actin promoter for which the use is likely to be similar in different epithelial cells of the same population. It is important to note that the level of expression of the transgene changes dramatically over time. Indeed, it frequently occurs that the amount of fluorescence of one animal goes from very high to very low within a few hours. This is probably because the expression of actin is regulated by factors affecting the entire animal (e.g., hormones) on a slow time scale. Therefore, time-lapse recordings were only performed when the level of fluorescence was high.

### NP sample, conA and NHS preparation

The streptavidin-coated Au NPs (#07211, Sigma-Aldrich, MO, USA) with a diameter of 40 nm were dispersed in 50 mM phosphate, 75 mM NaCl buffer, pH 7.4, with 20% glycerol (10–50 nM). Prior to use, the solution was centrifuged in a 0.22 µm-pore size centrifugal filter (Ultrafree, Millipore, MA, USA) to filter out large aggregates. For further dilution, the sample was centrifuged (6000 rpm, #6765/C1501, Corning, NY, USA) before every experiment for approximately 5 min until the supernatant was diluted to a light pink color (with an estimated concentration of <1 nM).

The sample with neutravidin-coated NPs (30 nm diameter, #C16A1-30-488-TN-50, Nanopartz, CO, USA) for the in vivo mice experiments (presented in Fig. 3e in the main text) was dispersed in 18 MEG DI water. NPs were diluted by adding 1 µl of the stock solution into 10 µl 1.1× ACSF_in vivo_ (containing in mM: 150 NaCl, 2.5 KCl, 10 HEPES, 2 CaCl_2_, and 1 MgCl_2_) to obtain a concentration of approximately 1 nM.

For the experiment conducted on the *Hydra*, streptavidin-coated Au NPs or Au NPs with a fluorescent tag (30 nm diameter, dispersed in 18 MEG DI water—#C16A1-30-488-TN-50, Nanopartz, CO, USA) were used for the incubation and gastric injection. The stock solution of fluorescently labeled Au NPs (16.6 nM) was diluted 10× with *Hydra* medium (in M: 1 CaCl_2_·H_2_O, 0.1 MgCl_2_·6H_2_O, 0.03 KNO_3_, 0.5 NaHCO_3_, and 0.08 MgSO_4_). For the streptavidin-coated NP-incubation, the filtered undiluted stock solution was used in a similar procedure. con A-biotin (#C2272, Sigma-Aldrich, MO, USA) was dissolved in Dulbecco’s phosphate-buffered saline (PBS) (Sigma-Aldrich, MO, USA) into a stock solution of ~200 µM. Prior to the experiments, the sample was diluted (in 1× PBS), sonicated and centrifuged in a 0.22 µm-pore size centrifugal filter (Ultrafree, Millipore, MA, USA), and sonicated again after centrifugation (~50 nM end concentration).

The NHS-biotin linker (#21312, Thermo Scientific, MA, USA) was dissolved in anhydrous dimethyl sulfoxide (DMSO) to a 250 mM stock solution. For the incubation, 2 µl was mixed with 300 µl ACSF solution and carefully pipetted onto the slice in the incubation chamber (total volume ~1 ml). The brain slice was then kept for 10 min at 35–37 °C under a 95% O_2_ and 5% CO_2_ atmosphere, after which the unincubated remainder of the compound was neutralized by rinsing the slice with PBS with 0.1% BSA.

For the absorption characterization (Fig. 1b), NPs were diluted in 1× PBS. A Perkin-Elmer Lambda 650 scanning UV-Vis spectrophotometer (PerkinElmer, OH, USA) was used.

### Electrophysiology

Patch-clamp recordings (pipette resistance ~4–8 MΩ) were obtained using pipettes pulled from borosilicate glass (1.5 mm and 1 mm OD, 0.86 mm and 0.5 mm ID—Sutter Instruments, Novato, CA, USA) using a DMZ puller (Zeitz-Instrumente GmbH, Munich, Germany) and established using a Multiclamp 700B amplifier (Molecular Devices, Union City, CA, USA). Electrical signals were acquired at 10 kHz (NI-DAQ BNC-2090, National Instruments, TX, USA) and Bessel-filtered at 4 kHz using a PC equipped with custom software (PackIO55, National Instruments, TX, USA) written in LabView (National Instruments, TX, USA) or, in case of the in vivo experiments, Prairie View 5.2 (Bruker, MA, USA). All electrophysiological slice recordings shown in this paper were obtained with a resting membrane potential between −65 mV and −75mV. For the slices, the external bath was continuously perfused with ACSF.

### Experimental procedure

#### Slices

Targeted stimulation experiments were achieved with a custom-made two-photon laser scanning microscope based on a modified Fluoview Olympus BX50WI microscope (Olympus, PA, USA) with a Ti:sapphire laser (Chameleon Ultra II, Coherent, CA, USA) as the excitation source and a ×40/0.8-NA objective (Olympus, PA, USA) to direct the excitation laser onto the sample. The laser power was modulated by a Pockels cell (Conoptics, CT, USA). The conA-biotin complex was locally perfused after achieving a whole-cell recording in close vicinity of the cell using a borosilicate pipette with a diameter of ~3 µm for a total duration of 2–3 min at a pressure of approximately 0.5 psi, manually applied with a syringe. Subsequently, the pipette was carefully retracted from the brain slice and replaced with a pipette filled with streptavidin-coated NP solution directed towards the cell. NPs were applied onto the cell under the same application conditions as the conA-biotin complex. Intracellular signals were recorded during the application processes to ensure that the health of the cell was not affected, and the electrical characteristics remained unchanged. Control experiments without NPs, but with the conA compound, confirmed that the application of the binding agent itself does not affect the AP threshold in response to the current injection. Stimulation patterns were either generated in custom-written LabView software^55^ as a cloud of point stimulations in a ring-like-shaped pattern (25−60 points, with a total duration of 50−100 ms) or using ScanImage (Vidrio Technologies, VA, USA) with spiral stimulations with a diameter of ~25 µm, 10−20 rotations, and a total duration of 10−50 ms, depending on the activation threshold of the neuron. Different focal planes were tested to achieve the best activation.

#### In vivo mouse experiments

During the entire experiment, the head-restrained animals were kept under isoflurane anesthesia (1% partial pressure in air) via a nose piece while their body temperature was maintained with a warming pad (37 °C). ACSF_in vivo_ was applied to the brain. A borosilicate pipette with an opening of approximately ~5 µm was filled with 50 nM conA in ACSF_in vivo_ containing 35 nM Alexa Fluor 488 Hydrazide. A micromanipulator (Sutter Instruments, Novato, CA, USA) was used to stir the pipette towards the exposed brain. A pressure of 2−5 psi was applied using a Picrospritzer III (Parker Hannifin, Hollis, NH) to expel conA-containing solution from the pipette. A two-photon microscope (Bruker, MA, USA) using a Mai Tai Deep See Ti:sapphire laser (Spectra Physics, Santa Clara, CA, USA) at 940 nm as the light source and a ×40/0.8 NA objective (Olympus) was used to monitor the fluorescence of Alexa Fluor 488 as the pipette penetrated the dura and was moved into the brain to a depth of approximately 160 µm. Pressure was increased, typically to 5−10 psi, to ensure that solution was expelled. After 10−15 min, the pipette was retracted. Next, a pipette with a ~4 µm opening was filled with NP solution in ACSF_in vivo_ containing 35 nM Alexa Fluor 488 and was inserted similarly to the first pipette in the vicinity (approximately 100 µm) of the injection site, using 0.5 psi pressure for 2−5 min, after which pressure was released. Pressure was controlled manually with a syringe. For the control experiments, only Alexa Fluor 488 in ASCF_in vivo_ was applied. The somata of neurons in layer 2 could be visualized as shadows due to the Alexa Fluor 488-containing NP solution being injected into the brain tissue. To perform loose-seal patch-clamp recordings, pipettes with a ~2 µm opening were filled with ACSF_in vivo_ containing 22 nM Alexa Fluor 594 Hydrazide. A second photomultiplier tube was used to visualize the patch-clamp pipette as extracellular recordings with a low resistance seal (typically 20−50 MΩ) were established targeting the shadows of neuron somata close to the NP-solution-containing pipette. To excite NPs, the same Chameleon Ultra II Ti:sapphire laser at 1040 nm used in the slice experiments was coupled into the microscope through a second set of galvanometer scanners. Here, the spiral characteristics were a diameter of ~25 µm, 20 rotations, and a spiral speed of 0.05 pix/µs (total duration of 630−960 ms—generated in PrairieView 5.2, Bruker, MA, USA). Prior to the consecutive stimulations, different focal planes were tested until neuronal activity was observed.

### Hydra in vivo experiments

*Hydra* were soaked in NP-containing medium in microwells (50 µl per well) and transferred to a regular well prior to the experiment. For the gastric injection, individual *Hydra* were tapped on the foot with forceps to stimulate contraction, and the NP solution was manually injected with a microinjection (with the same pipettes and procedure as the injection of the *Hydra* oocytes—see section “Generation of transgenic *Hydra*”). The *Hydra* were carefully monitored several days prior and post NP incubation to ensure their viability. To restrict spontaneous muscle contractions, the NP-incubated *Hydra* were sedated, and their mobility was restricted by (1) anesthetics with 200 µM of the myosin II ATPase inhibitor 4-methyl-*N*-(phenylmethyl)benzenesulfonamide (#1576-37-0, Tocris, Bristol, UK) for 1 h prior to the experiment and (2) placement in a thin layer of 1% agarose in between two microscope coverslips, confining their mobility for high-accuracy stimulation and imaging. For the most part, the experiments were carried out with the same microscope setup as for the mouse in vivo experiments. Imaging was performed using a 20x/0.5NA objective (Olympus, PA, USA) at a frame rate of 1.54 fps. The excitation parameters were 100 µm diameter spirals in the case of systemic excitation, and ~25 µm diameter for the stimulation of individual cells, 14 rotations, and a spiral speed of 0.01 pix/µs (total duration of ~1200−1500 ms). Bright-field images were taken with a Leica microscope setup (M165FC, Leica, IL, USA) with a scientific CMOS camera (Orca-Flash4.0, Hamamatsu, Japan) at 10x magnification.

### Linear and SH nonlinear contributions to the absorption of excitation energy by Au NPs

To quantify the total absorbed energy of the excitation laser by the Au NPs, we first evaluated the linear contribution at the NIR excitation wavelength. For sufficiently high excitation power, this contribution is expected to be less significant than the nonlinear component, as the plasmon resonance feature for these sizes of NPs lies in the visible regime of the spectrum (see Fig. 1b). The (linear) absorption cross-section $C_{{\rm {abs}}}^{({\rm {NIR}})}$ of an Au NP excited by an NIR plane wave with electric field amplitude $E_0^{({\rm {NIR}})}$ and wavenumber $k_0^{({\rm {NIR}})}$ is defined as:

3 $$C_{{{\rm abs}}}^{({{\rm NIR}})} = k_0^{({{\rm NIR}})}{\int\int\int}_V {\varepsilon _2^{({ {\rm NIR}})}\frac{{|E^{({{\rm NIR}})}|^2}}{{|E_0^{({{\rm NIR}})}|^2}}{\rm d}V}$$

where *V* is the volume of the NP, $\varepsilon _2^{({\rm {NIR}})}$ is the imaginary permittivity of gold (the imaginary part takes into account electron damping by scattering processes, by which heat is generated), and $E^{({\rm {NIR}})}$is the electric field amplitude across the NP. All quantities are relative to the NIR wavelength 1040 nm. We calculate $E^{({\rm {NIR}})}$ using the standard model of Mie scattering from homogeneous spherical particles. Since $E^{({\rm {NIR}})}$ is proportional to $E_0^{({\rm {NIR}})}$, $C_{{\rm {abs}}}^{({\rm {NIR}})}$ is independent of the excitation power. It is useful to note that $C_{{\rm {abs}}}^{({\rm {NIR}})}$ is significantly smaller than it would be if the excitation was in the visible spectrum because $\varepsilon _2^{({\rm {NIR}})}$ is negligible at the NIR excitation wavelength.

The SH absorption cross-section $C_{{\rm {abs}}}^{({\rm {SH}})}$ is defined analogously in Eq. 1 of the main text. In contrast to $C_{{\rm {abs}}}^{({\rm {NIR}})}$, $C_{{\rm {abs}}}^{({\rm {SH}})}$ grows quadratically with $|E_0^{({\rm {NIR}})}|$ because $|E^{({\rm {SH}})}| \propto |E_0^{({\rm {NIR}})}|^2$. In addition, $C_{{\rm {abs}}}^{({\rm {SH}})}$ grows quadratically with the second-order bulk and surface susceptibilities.

To translate the absorption model into a local temperature increase, we use the standard definition of heat capacity:

4 $$E_{{\rm {abs}}} = m_{{\rm {NP}}}c_{{\rm {NP}}}\Delta T$$

where $E_{{\rm {abs}}}$ is the absorbed energy in the NP, $m_{{\rm {NP}}}$ is the mass of an NP ($m_{{\rm {NP}}} = V_{{\rm {NP}}}\rho _{{\rm {NP}}}$ where $\rho _{{\rm {NP}}}$ is the mass density) and $c_{{\rm {NP}}}$ is the specific heat capacity of gold (=0.129 Jg^−1^ K^−1^) at room temperature. The temperature increases above the experimental activation threshold in the NP (indicated by the dotted line in Fig. 5b) are ≳20 °C (at the surface of the NP). Henceforth, we can determine the temperature increase at the cell membrane by using the approximative expression for the spatial temperature dependence for (ultrashort) pulsed excitation^25^:

5 $$T(r) = \frac{{E_{{\rm {abs}}}}}{{3\sqrt 3 c_{\rm {w}}\rho _{\rm w}r^3}}$$

where $c_{\rm w}$is the heat capacity of water (4.187 Jg^−1^ K^−1^), $\rho _{\rm w}$ is the mass density, and *r* is the distance from the NP surface.

## Electronic supplementary material

Supplementary Material

Full time-dependent recording of activity in Hydra (1)

Full time-dependent recording of activity in Hydra (2)

## Data Availability

The datasets generated during the current study are available from the corresponding authors on reasonable request.
