# Supplementary material for: Neuronal photoactivation through second-harmonic near-infrared absorption by gold nanoparticles
Source: Light Sci Appl. 2018 Dec 5;7:100. doi: 10.1038/s41377-018-0103-0 (PMC6279767; doi:10.1038/s41377-018-0103-0)
Supplement: Supplementary file 1 — Supplementary Material [file 41377_2018_103_MOESM1_ESM.pdf]

Neuronal photoactivation through second-harmonic near-infrared absorption by gold nanoparticles

Wieteke D. A. M. de Boer[†,*,1], Jan J. Hirtz[†,*,1], Antonio Capretti[2], Tom Gregorkiewicz[2], Mercè Izquierdo-Serra[1], Shuting Han[1], Christophe Dupre[1], Yuriy Shymkiv[1] and Rafael Yuste[1]

[†] these authors contributed equally to the work

[1]NeuroTechnology Center, Department of Biological Sciences, Columbia University, New York, New York 10027, USA.

[2]Van der Waals – Zeeman Institute, University of Amsterdam, 1098 XH Amsterdam, The Netherlands

*Corresponding Authors

Supplementary Material

Full time-dependent recording of activity in Hydra (1).

Movie demonstrating the increase in GCaMP fluorescence and contraction activity of Au NP-incubated Hydra in response to photostimulation, corresponding to Figure 4 c,d. The speed of the recording is set to two times original speed. White dots in the upper right corner mark the times of photostimulation.

Full time-dependent recording of activity in Hydra (2).

Movie demonstrating the increase in GCaMP fluorescence of single epithelial muscle cells of Au NP-incubated Hydra in response to photostimulation, corresponding to Figure 4 f,g. The speed of the recording is set to two times original speed. White dots in the upper right corner mark the times of photostimulation.
